# Ocular Manifestation of *CACNA1A* Pathogenic Variants

**DOI:** 10.15844/pedneurbriefs-30-12-2

**Published:** 2016-12

**Authors:** Karit Reinson, Katrin Õunap

**Affiliations:** 1 Department of Clinical Genetics, United Laboratories, Tartu University Hospital, Tartu, Estonia; 2 Department of Paediatrics, Institute of Clinical Medicine, University of Tartu, Tartu, Estonia

**Keywords:** *CACNA1A*, Ocular Manifestation, Global Developmental Delay

## Abstract

Investigators from The Children’s Hospital at Westmead in New South Wales; The Queensland University of Technology in Brisbane; Sydney Children’s Hospital in New South Wales and Laboratoire de Genetique in Paris investigated children with a proven heterozygous missense pathogenic variant in the *CACNA1A* gene.

Investigators from The Children’s Hospital at Westmead in New South Wales; The Queensland University of Technology in Brisbane; Sydney Children’s Hospital in New South Wales and Laboratoire de Genetique in Paris investigated children with a proven heterozygous missense pathogenic variant in the *CACNA1A* gene. The *CACNA1A* gene encodes the alpha-1 subunit of the voltage-gated calcium channel. Expression of these channels is particularly high in neuronal tissue, especially in the cerebellum. The literature on *CACNA1A* disorders in children is relatively modest, and the focus of the range of ocular presentations in childhood remains rare. The authors reviewed retrospectively nine children from Children’s Hospital at Westmead over a 10-year period (2005–2015). All of them had confirmed heterozygous mutation in the *CACNA1A* gene. Eye movement disorders like paroxysmal tonic upgaze (PTU), strabismus, and abnormal saccades were the presenting feature in eight of the nine children. There was a wide range in the age of presentation of the first sign (2mo–10y), though six of the nine children demonstrated the eye movement disorder in the first 2 years of life. None of them followed a ‘benign’ course. The children presenting with ocular abnormalities had additional problems including hypotonia, cerebellar ataxia, or epilepsy. Six patients were diagnosed with global developmental delay within 2 years of their initial presentation, including all three patients with PTU. In total, 5 patients had an abnormal brain MRI - cerebellar or generalized mild cerebral atrophy. Based on the previously described findings, the authors suggest that an eye movement disorder may be a clue to the underlying mutation in the *CACNA1A* gene, especially if there is evidence of developmental delay or cerebellar or cerebral atrophy on MRI. [1]

COMMENTARY. This interesting overview of children with heterozygous missense pathogenic variants in the *CACNA1A* gene gives a new perspective on the disease course. Since the concept of a ‘pre-symptomatic’ eye movement disorder was previously described in children [2, 3] and adults diagnosed with SCA6 [4], the suggestion that all children with PTU, and an ocular motor apraxia or strabismus (especially when associated with developmental delay or cerebellar atrophy), should be considered for *CACNA1A* genetic testing.

Importantly, a study like this calls attention to the wide phenotypic spectrum of patients with *CACNA1A* mutations. Moreover, we have recently described two sibs with bi-allelic *CACNA1A* pathogenic variants, which cause early onset epileptic encephalopathy, cerebral, cerebellar atrophy and optic nerve atrophy [5]. All this additional information could lead to better counselling regarding the prognosis at the time of diagnosis (e.g. episodes of severe hemiplegic migraine) as well as implementing more targeted therapies like verapamil [6].

As the authors pointed out, the weakness of their study is that it is retrospective with small number of patients and quite short period of follow-up. A multicenter research study with gene sequencing of all children with aforementioned eye movement disorders would identify the true frequency of the *CACNA1A* pathogenic variants in this cohort.
